# Neck muscle cross-sectional area, brain volume and cognition in healthy older men; a cohort study

**DOI:** 10.1186/1471-2318-13-20

**Published:** 2013-02-28

**Authors:** Alixe HM Kilgour, Karen J Ferguson, Calum D Gray, Ian J Deary, Joanna M Wardlaw, Alasdair MJ MacLullich, John M Starr

**Affiliations:** 1 Centre for Cognitive Ageing and Cognitive Epidemiology, The University of Edinburgh, 7 George Square, EH8 9JZ, Edinburgh, UK; 2 Geriatric Medicine Unit, University of Edinburgh, Edinburgh, UK; 3 Clinical Research Imaging Centre, Queen’s Medical Research Institute, University of Edinburgh, Edinburgh, UK; 4 Department of Psychology, University of Edinburgh, Edinburgh, UK; 5 Brain Research Imaging Centre, Clinical Neurosciences, University of Edinburgh, Edinburgh, UK; 6 Scottish Imaging Network, A Platform for Scientific Excellence (SINAPSE), Edinburgh, UK

**Keywords:** Sarcopenia, Cognition, Aging, Muscle cross-sectional area, Brain volume

## Abstract

**Background:**

Two important consequences of the normal ageing process are sarcopenia (the age-related loss of muscle mass and function) and age-related cognitive decline. Existing data support positive relationships between muscle function, cognition and brain structure. However, studies investigating these relationships at older ages are lacking and rarely include a measure of muscle size. Here we test whether neck muscle size is positively associated with cognition and brain structure in older men.

**Methods:**

We studied 51 healthy older men with mean age 73.8 (sd 1.5) years. Neck muscle cross-sectional area (CSA) was measured from T1-weighted MR-brain scans using a validated technique. We measured multiple cognitive domains including verbal and visuospatial memory, executive functioning and estimated prior cognitive ability. Whole brain, ventricular, hippocampal and cerebellar volumes were measured with MRI. General linear models (ANCOVA) were performed.

**Results:**

Larger neck muscle CSA was associated with less whole brain atrophy (t = 2.86, p = 0.01, partial eta squared 17%). Neck muscle CSA was not associated with other neuroimaging variables or current cognitive ability. Smaller neck muscle CSA was unexpectedly associated with higher prior cognition (t = −2.12, p < 0.05, partial eta squared 10%).

**Conclusions:**

In healthy older men, preservation of whole brain volume (i.e. less atrophy) is associated with larger muscle size. Longitudinal ageing studies are now required to investigate these relationships further.

## Background

As the population of the world ages, governments, research funding bodies and the general public are becoming increasingly interested in promoting healthy ageing. Healthy ageing is not just the avoidance of pathology but also the slowing down of the natural rate of ageing, mainly through lifestyle adaptations. Two important consequences of the normal ageing process are sarcopenia and age-related cognitive decline (ARCD).

Sarcopenia is the loss of muscle mass and function with advancing age [1-3]. It is a main component of the frailty syndrome, and greater degrees of sarcopenia are associated with increased levels of falls, disability, morbidity and death [4-6]. Muscle mass is lost from the third decade at a rate of 1-2% per year, increasing with age [7-9]. Muscle function deteriorates more quickly, with studies showing strength to decline by 1-4% per year and power to decline by 3-4% per year [8,10]. ARCD is the normal and universal change in cognition seen with increasing age [11]. Such changes mainly affect the so-called ‘fluid’ abilities (eg working memory, speed of processing, reasoning). Crystallised abilities (eg. vocabulary, knowledge, autobiographical memory) remain largely intact [12,13]. Studies have shown that the decline seen in fluid cognitive abilities begins in early adulthood, with some deterioration seen by the early 20s [14].

Until recently, muscle and brain ageing had rarely been studied in tandem; however, studies demonstrating improved cognition in later life with increased physical activity have highlighted this important area of research. For example a Cochrane review of randomised controlled trials found evidence that aerobic physical activities improved cognitive function in healthy older adults, with effects observed for motor function, cognitive speed, and auditory and visual attention [15]. Observational studies have also found positive relationships between physical activity and cognitive function [16,17]. There is some evidence that both grey and white matter brain volume can be significantly improved by aerobic exercise in older adults [18]. It was hypothesized that these relationships were due solely to cardiovascular fitness, and although this may play a role, animal studies have found other underlying mechanisms including: increased levels of brain-derived neurotrophic factor which may contribute to neurogenesis, effects on neurotransmitter systems and increased insulin-like growth factor 1 [19].

Several studies have also demonstrated a positive relationship between muscle function (eg handgrip strength, gait speed) and cognition [20-23]. Possible mechanisms which might account for the shared variance in muscle and brain size and function with healthy ageing include: the role of hormones and growth factors (eg glucocorticoids) [24,25]; immunosenescence and inflammation (eg IL6 and CRP) [26,27]; oxidative stress and mitochondrial ageing [28,29]; decreased stem cell activity [30,31]; and environmental and lifestyle factors (eg smoking) [32,33].

The above findings showing association between brain and muscle structure and function add support to the common cause hypothesis, that core underlying processes determine the rate of ageing in each organ throughout the body. If we are able to demonstrate an association between muscle size and brain size and function, this could add further weight to the common cause hypothesis [34-37]. This would have large implications for future treatments designed to modify the rate of ageing, which could possibly target several organs at once (eg muscle and brain) as opposed to individualised treatments being developed.

It is known that muscle size and function (ie strength or power) do not age in parallel [10,38], therefore the association between muscle size and either brain structure (eg whole brain volume) or cognitive function requires independent study. We found only one study which has investigated the relationship between muscle bulk and brain structure in older adults; however, this study included subjects with Alzheimer’s disease along with normal controls [39]. The studies investigating muscle size and cognition have largely relied on simple cognitive screening tools (eg Mini Mental State Examination (MMSE)) and do not contain a measure or estimate of prior cognitive ability [40-42].

Here we studied community-dwelling healthy older men, measuring: neck muscle cross-sectional area (CSA), multiple cognitive domains including estimated prior cognitive ability and neuroimaging volumes. We hypothesised that lower muscle bulk is associated with structural markers of brain ageing, and poorer cognitive ability in healthy older people.

## Methods

### Participants

Participants were 51 community-dwelling men involved in a longitudinal ageing study investigating healthy ageing, glucocorticoid status and brain structure [43,44]. The study was approved by the Lothian Health Ethics Committee. All subjects gave written informed consent and the research was carried out in compliance with the Helsinki Declaration. Data from the second wave of the study were used because the smaller MR head coil used in the first wave excluded the neck muscles. Exclusion criteria were previously provided [43,44]. Participants were healthy, lacking of significant illness, including dementia, stroke, ischaemic heart disease, depressive illness, excessive alcohol intake, and cancer. No participants were taking psychotropic medication.

### MR brain imaging

The full MR brain imaging protocol has been previously published [43]. In summary, imaging was performed on a GE Signa LX 1.5 T (General Electric) MR scanner. Participants received a sagittal T_1_-weighted spin echo sequence covering the whole head (TR 450 msec, TE 9 msec, FOV 24 cm, matrix 256 × 224, slice thickness 5 mm (no gap)) and a volume scan consisting of a T_1_-weighted 3D inversion recovery prepared sequence (3D IR_PREP) acquired in the coronal plane with slices perpendicular to the long axis of the hippocampus and covering the whole head (TI 600 msec with TE set to minimum, FOV 22 cm, matrix 256 × 192, slice thickness 1.7 mm (no gap)).

### Brain structure measurements

Image analysis was performed using Analyze v7.0 for Windows (Mayo Clinic, Rochester, MA). Whole brain, hippocampal and ventricular volumes [43], and intracranial area (a validated estimate of intracranial volume [45]) were obtained by an experienced rater.

### Neck muscle cross-sectional area

We used neck muscle cross-sectional area (CSA) as a measure of muscle size. We have previously shown in a study of 24 subjects that neck muscle CSA is strongly correlated with thigh muscle CSA (R^2^ 0.77), which is often used as a proxy for general muscle bulk [46]. Neck muscle CSA is generally available in brain MRI studies, whereas thigh muscle CSA is not usually measured within longitudinal cognitive ageing studies. Neck muscle CSA was measured using a validated technique [46]. Full details can be obtained in the technique paper; however, in summary, the mid-point of the C2-vertebra was located in the sagittal slice of a 3D reconstructed image. The image was then converted to a transverse view and the posterior neck muscles were outlined using a cursor. The software then calculated the contained area. The muscles measured were the semispinalis capitis, splenius capitis and trapezius (measured as a combined group), and the sternocleidomastoid.

### Tests of cognitive function

The following tests of cognitive function were performed as part of the original study, as previously described [43]: the Mini-Mental State Examination (MMSE, a screening test for cognitive impairment); the Controlled Word Association Test (CWAT, tests verbal fluency, which is an aspect of executive function); the Digit-Symbol Substitution Test (DSST, tests attention and processing speed) from the Wechsler Adult Intelligence Scale; Raven’s Standard Progressive Matrices (RSPM, tests non-verbal reasoning, an important aspect of fluid intelligence); Logical Memory (tests immediate and delayed verbal declarative memory); Visual Reproduction (tests immediate and delayed visual memory); Rey’s Auditory-verbal Learning Test (tests verbal memory and learning); Benton’s Visual Retention Test (tests visual memory); and the National Adult Reading Test (NART, provides an estimate of prior general cognitive ability). Due to the strong correlation between pre-morbid cognitive ability (of which NART provides an estimate) and educational achievement [47], it was decided to include only NART and not educational achievement as a predictor variable. One participant had a MMSE below 24 and was excluded from the analysis as this score may be reflective of an incipient diagnosis of dementia.

### Statistical analysis

Descriptive statistics, exploratory analyses, general linear modeling (Analysis of Covariance; ANCOVA) and principal components analysis were performed on SPSS version 18.0 for Windows. Missing values were excluded listwise. For the ANCOVA we constructed baseline models with the measures of brain structure and cognitive ability as dependent (i.e. outcome) variables and neck muscle CSA as an independent variable, adjusting for intracranial area (ICA) and age. ICA is thought not to change after the onset of age-related neuronal loss, particularly in men, therefore can be used as a marker of peak brain size, thus allowing the outcome variable to be more reflective of brain atrophy [48]. Also, without adjusting for ICA it could be argued that those with bigger skulls require larger neck muscles for support or that they are just larger in proportion; therefore, this adjustment also controls for this. We also adjusted for NART since this was found to correlate significantly with neck muscle CSA, brain volumes and current cognition. Furthermore, in the models with current cognitive ability as an outcome variable, adjusting for the NART score allows the model to reflect the degree of cognitive ageing that has taken place.

## Results

All participants (n = 51) were male with a mean age of 73.8 years (sd 1.5). Descriptive statistics for participant neuroimaging and neck muscle cross-sectional area data are in Table 1. Of the 51 MR brain scans reviewed, we were unable to measure neck muscle CSA on one scan as the scan did not extend far enough inferiorly to include the neck muscles at the required level for measurement (the mid-point of the C2-vertebra). In a further two cases, the hippocampal volume could not be measured.

**Table 1:** Neuroimaging and muscle cross-sectional area data

	**N**	**Mean**	**Std. Deviation**
Whole brain volume (cm^3^)	50	1149.8	90.4
Intracranial area (cm^2^)	50	157.9	9.6
Total ventricular volume (cm^3^)*	50	31.2	24.0
Total hippocampal volume (cm^3^)	48	7.0	0.8
Cerebellar volume (cm^3^)	50	135.4	14.3
Total SCM muscle area (cm^2^)	49	5.1	1.1
Total comb muscle area (cm^2^)	49	22.8	3.1
Total muscle area (cm^2^)	49	28.0	3.4
Valid N (listwise)	47		

*Non-parametric data, median and inter-quartile range presented.

Table 2 contains the descriptive statistics for the cognitive test data. To reduce the risk of type 1 statistical error by testing multiple associations, we performed principal components analysis. The Kaiser-Meyer-Olkin measure verified sampling adequacy. Two principal components were extracted employing varimax rotation; the first had an eigenvalue of 2.58, explaining 36.9% of the variance and the second had an eigenvalue of 2.4, explaining 34.3% of the variance. For comprehensibility, we refer to these components as ‘factors’ as is general usage. Together, the factors explain 71.2% of the variance in the cognitive test scores. The cognitive tests which focused on memory (ie Logical Memory, Visual Reproduction, AVLT and BVRT) had high factor loadings for Factor 1; therefore, we hereafter call this factor the Memory Factor. Factor 2 had high factor loadings for the three other tests: CWAT, DSST and RSPM and hereafter is referred to as the Cognitive Processing Factor.

**Table 2:** Prior and current cognition data

	**N**	**Mean**	**Std. Deviation**
National adult reading test *	47	42.0	13.0
Mini mental state Examination *	50	28.0	4.0
Controlled word association test	47	40.4	10.9
Digit symbol substitution test	46	43.3	10.7
Raven’s standard progressive matrices	46	40.0	9.1
Logical memory	47	51.6	15.0
Visual reproduction	47	46.4	14.8
Auditory-verbal learning test	47	49.9	9.0
Benton visual retention test *	47	17.0	4.0
Factor 1 (Memory)	45	0.0	1.0
Factor 2 (Cognitive processing)	45	0.0	1.0
Valid N (listwise)	45		

*Non-parametric data, median and inter-quartile range presented.

Initially bivariate statistics were performed (Spearman’s rho) (Table 3). The only variable to significantly correlate with total neck muscle CSA was NART (rho -.36, p = .01). NART was also found to correlate strongly with the following variables: intracranial area; unadjusted whole brain, hippocampal, and cerebellar volumes; and the cognitive processing factor.

**Table 3:** Spearman’s rho correlations between neuroimaging, muscle and cognitive variables (with p values)

	**Total neck muscle CSA**	**NART**
Age	.21	-.03
	(.15)	(.82)
Whole brain volume	.07	.49
	(.62)	(<0.001)
Intracranial area	-.11	.56
	(.44)	(<0.001)
Total ventricular volume	.13	.10
	(.10)	(.50)
Hippocampal volume	-.07	.48
	(.67)	(<0.001)
Cerebellar volume	.03	.46
	(.83)	(<0.001)
Memory factor	-.28	.13
	(.07)	(.40)
Cognitive processing factor	-.03	.52
	(.83)	(<0.001)
NART-	-.36	1.00
	(.01)	

ANCOVA was performed to check for shared variance among neck muscle CSA and the neuroimaging measures, the cognitive factors, and NART. Models were corrected for age, intracranial area (ICA, to correct for head size) and NART, except in the model for NART where only age and ICA were adjusted for. Total neck muscle CSA was found to predict 17% of the variance in whole brain volume (t = 2.86, p = 0.01) (Table 4). However, total neck muscle CSA did not significantly predict the variance in ventricular, hippocampal or cerebellar volumes (p > 0.05).

**Table 4:** ANCOVA for whole brain volume with total neck muscle area, intracranial area, age and prior cognition as predictor variables

**Source**	**Degrees of freedom**	**F**	Unstandardised B*	**t**	**Sig.**	**Partial Eta squared**
**Corrected model**	4	12.85			<.01	.56
**Intercept**	1	1.06	491.40	1.03	.31	.03
**Age (years)**	1	0.79	-5.64	-.89	.38	.02
**Intracranial area (cm**^**2**^**)**	1	13.13	0.04	3.62	<.01	.24
**NART (Score out of 50)**	1	8.43	3.85	2.90	.01	.17
**Total neck muscle CSA (cm**^**2**^**)**	1	8.16	0.09	2.86	.01	.17
**Corrected total**	45					

*Unstandardised B coefficients reflect change in whole brain volume in cm^3^.

Neck muscle CSA did not significantly predict variance in either the memory factor or the cognitive processing factor (p > 0.05) (Tables 5 &6). Using the NART score as an outcome variable, we found that total neck muscle CSA predicts 10% of the variance in the NART score (t = −2.12, p < 0.05) after adjusting for ICA and age.

**Table 5:** ANCOVA for Memory factor with total neck muscle area, intracranial area, age and prior cognition as predictor variables

**Source**	**Degrees of freedom**	**F**	Unstandardised B*	**t**	**Sig.**	**Partial Eta squared**
**Corrected model**	4	1.23			.31	.11
**Intercept**	1	1.39	8.91	1.18	.25	.03
**Age (years)**	1	1.30	-.12	-1.14	.26	.03
**Intracranial area (cm**^**2**^**)**	1	0.02	<-.01	-.15	.88	.00
**NART (Score out of 50)**	1	1.56	.03	1.25	.22	.04
**Total neck muscle CSA (cm**^**2**^**)**	1	0.28	<.01	-.53	.60	.01
**Corrected total**	43					

*Unstandardised B coefficients reflect change in factor score for the Memory Factor.

**Table 6:** ANCOVA for General Processing Factor with total neck muscle area, intracranial area, age and prior cognition as predictor variables

**Source**	**Degrees of freedom**	**F**	Unstandardised B*	**t**	**Sig.**	**Partial Eta squared**
**Corrected model**	4	5.19			<.01	.35
**Intercept**	1	0.23	3.06	.48	.63	.01
**Age (years)**	1	1.60	-.11	-1.26	.21	.04
**Intracranial area (cm**^**2**^**)**	1	0.15	<.01	.39	.70	.00
**NART (Score out of 50)**	1	11.86	.06	3.44	<.01	.23
**Total neck muscle CSA (cm**^**2**^**)**	1	2.86	<.01	1.69	.10	.07
**Corrected total**	43					

*Unstandardised B coefficients reflect change in factor score for the General Processing Factor.

## Discussion

We found that in healthy elderly men, preservation of whole brain volume was associated with larger total neck muscle cross-sectional area. Therefore in an elderly cohort, those that have a smaller muscle bulk have undergone more brain atrophy. This finding supports the common cause hypothesis, by demonstrating that the rate of sarcopenia and ARCD may occur in parallel within individuals, driven by core underlying biological processes. However, we found no significant association between total neck muscle CSA and ventricular volume (a different measure of brain atrophy), or hippocampal or cerebellar volumes.

We unexpectedly found that total neck muscle CSA was significantly negatively associated with estimated prior cognitive ability (NART) after adjustment for ICA and age, but we found no significant association between total neck muscle CSA and current cognitive abilities. This suggests that those with lower prior cognitive ability may have larger muscles in old age. Muscle mass in old age is determined by 2 factors. Firstly, peak muscle bulk obtained in young adulthood, and secondly, rate of muscle atrophy with ageing. Therefore we hypothesise that those with lower cognitive abilities may have undertaken more manual work [49,50] and therefore achieved a greater peak muscle bulk and a larger muscle mass in old age. We can find no plausible explanation as to why a lower prior cognitive ability would favour a slower rate of muscle atrophy. We unfortunately do not have sufficiently detailed previous occupational history or socio-economic class data for the participants to be able to test this theory further at this point.

We found only one previous study which investigated the relationship between muscle size and brain size, and this study also found a positive relationship between muscle bulk and whole brain volume. Burns et al. studied elderly people with early Alzheimer’s disease (AD) (n = 70) or normal cognition (n = 70) and found that whole brain volume, normalized for head size, was predictive of lean mass as measured by DEXA (Beta .20, p < .001) in both groups [39]. White matter volume was the primary driving factor for the relationship (Beta .19, p < .001) while grey matter volume showed no association with lean mass. This indicates that the cause of loss of lean muscle mass in AD may be different to normal ageing as it is primarily grey matter that is lost in AD.

In the above study Burns et al. also investigated the relationship between MMSE and a measure of global cognitive performance (a composite score made up of the results of a battery of tests, including the DSST and verbal fluency) with muscle mass [15]. They found a significant positive association between both the global cognitive performance score (Beta .12, p = .007) and MMSE (Beta .11, p = .009) and muscle mass, controlling for age and sex but not for prior cognition which we have shown to correlate with both brain and muscle size (Table 3). Our study was able to investigate the relationship between cognitive decline, by adjusting for prior cognition using the NART score, and current cognition, whereas this study only looked at cross-sectional data from current cognition. This may explain why they found an association between current cognition and muscle mass and we did not.

Several large studies have also investigated the links between muscle size and cognition. In a large cross-sectional study of community dwelling women aged 75 or over (n = 7105), Nourhashemi et al. found that low cognitive function was associated with low fat free mass [41]. However the cognitive test used was the Short Portable Mental Status Questionnaire (SPMSQ), which consists of only 10 questions and is mainly used as a screening test for cognitive impairment.

Conversely, Wirth et al. studied 4095 consecutive geriatric hospital patients and found that fat-free mass was not associated with cognitive dysfunction, measured using MMSE, after adjusting for age, sex and Barthel index [42]. Also, Auyeung et al. studied 2737 cognitively normal older people and found that appendicular skeletal muscle mass (ASM) was significantly predictive of MMSE 4 years later in men but not women [40]. However, after adjustment for age, years of education and baseline MMSE score, the relationship in men was not significant either.

Our study has the benefit of including tests of both prior and current cognitive function. This allows us to look at cognitive decline rather than purely at current cognitive ability, and is the only study we could find that specifically tested the relationship between prior cognition and muscle size. Also, the three large studies mentioned above used cognitive tests which are primarily designed to screen for cognitive impairment (ie SPMSQ and MMSE) rather than to detect the subtleties of change in cognition with age [40-42], for which our cognitive tests were specifically chosen. Burns et al. used more detailed cognitive tests; however the numbers involved in their study are much smaller compared to the other three studies. Our study is the first to measure muscle cross-sectional area and cognition or brain size; the above mentioned studies used either bioimpedence analysis or DEXA as the measure of muscle bulk.

The main limitations of our study are the lack of longitudinal data and the small sample size. In ageing studies longitudinal data are crucial as it is the rate of loss of muscle size or brain size that is of interest rather than measurements at a cross-sectional time point. With brain size we can partially correct for this using intracranial area, but with muscle size we are unsure if someone has lost 10% of their lean body mass in the previous decade or 50%, as clearly the peak muscle bulk obtained will affect the final outcome greatly. The study also contained mainly white males and this will affect the generalisability of our results.

## Conclusion

In healthy older men preservation of whole brain volume is associated with larger muscle size and larger muscle size was associated with lower prior cognition, but not current cognition. These results support previous work in this area which has also found associations between brain and muscle variables in older adults. Longitudinal ageing studies are now required to investigate these relationships further.

## Competing interests

The authors have declared that no competing interests exist.

## Authors’ contributions

Conceived and designed the project: AK IJD JMW AMJM JMS. Performed the measurements: AK, KJF, AMJM. Analyzed the data: AK IJD JMW AMJM JMS. Contributed analysis tools: CDG. Wrote the paper: AK KJF CDG IJD JMW AMJM JMS. All authors read and approved the final manuscript.

## Pre-publication history

The pre-publication history for this paper can be accessed here:

http://www.biomedcentral.com/1471-2318/13/20/prepub
